# Mechanisms underlying the virulence regulation of new *Vibrio alginolyticus* ncRNA Vvrr1 with a comparative proteomic analysis

**DOI:** 10.1080/22221751.2019.1687261

**Published:** 2019-11-12

**Authors:** Yanfei Zuo, Lingmin Zhao, Xiaojin Xu, Jiaonan Zhang, Jiaolin Zhang, Qingpi Yan, Lixing Huang

**Affiliations:** a Fisheries College, Key Laboratory of Healthy Mariculture for the East China Sea, Ministry of Agriculture, Jimei University, Xiamen, PR People’s Republic of China; b Key Laboratory of Special Aquatic Feed for Fujian, Fujian Tianma Technology Company Limited, Fuzhou, People’s Republic of China

**Keywords:** NcRNA, *pykf*, *Vibrio alginolyticus*, virulence, Vvrr1

## Abstract

The incidence of *Vibrio alginolyticus* infections has increased in recent years due to the influence of climate change and rising sea temperature. Vibrio virulence regulatory RNA 1 (Vvrr1) is a newly found noncoding RNA (ncRNA) predicted to be closely related to the adhesion ability of *V. alginolyticus* based on the previous RNA-seq. In this study, the target genes of Vvrr1 were fully screened and verified by constructing Vvrr1-overexpressing strains and using the proteome sequencing technology. Pyruvate kinase I (*pykF*) gene was predicted to be a chief target gene of Vvrr1 involved in virulence regulation. The adhesion ability, biofilm formation and virulence were significantly reduced in the Vvrr1-overexpressing and the *pykF*-silenced strain compared with the wild strains. Similar to the overexpression of Vvrr1, the silencing of pykF also reduced the expression level of virulence genes, such as *ndk*, *eno*, *sdhB*, *glpF*, and *cysH*. Meanwhile, by constructing the “*pykF-GFP*” fusion expression plasmid and using the GFP reporter gene analysis in *Escherichia coli*, the fluorescence intensity of the strain containing Vvrr1 whole ncRNA sequence vector was found to be significantly weakened. These indicated that Vvrr1 participated in the virulence regulation mechanism of *V. alginolyticus* by interacting with the virulence gene *pykF*.

## Introduction

*Vibrio alginolyticus*, a gram-negative halophilic bacterium distributed worldwide [1], is a normal inhabitant of coastal and estuarine environments in warm tropical regions. It also represents one of the leading opportunistic pathogens for people who consume raw or undercooked contaminated seafood or engage in water activity. It may cause severe soft tissue infections, sepsis, and other extraintestinal infections [2–7]. *V. alginolyticus* is one of roughly a dozen *Vibrio* species that cause human illness and signiﬁcant morbidity (multiple surgeries, prolonged antibiotic treatment, amputations, and other complications can occur) and mortality [8,9]. The incidence of *V. alginolyticus* infections has increased, perhaps owing in part to the spread of *V. alginolyticus* favored by climate change and rising seawater temperature [10]. *V. alginolyticus* infections were reported to the Cholera and Other Vibrio Illness Surveillance (COVIS) system in the USA since 1988. The surveillance started in the Gulf Coast states and has expanded over time, and *Vibrio* infection became nationally notiﬁable in 2007. An analysis of both COVIS data and data from the 10-state Foodborne Diseases Active Surveillance Network (FoodNet) from 1996 to 2010 showed a dramatic increase in the incidence of *V. alginolyticus* infection in both systems [2]. Using data from 1988 to 2012, Newton et al. also categorized infections using specimen source and exposure history, analyzed case characteristics, and calculated the incidence rates using the US Census Bureau data [11]. The reported incidence of infections increased 12-fold over the study period, although the extent of diagnostic or surveillance bias was unclear. For many years, *V. alginolyticus* was the third most common *Vibrio* species reported in human illness, but it has been the second most common *Vibrio* species since 2007 due to increasing overall vibriosis rates, despite substantial increases in other species [12]. Considering that *V. alginolyticus* is an emerging microbe that causes severe infections in both humans and marine animals [13,14], a better understanding of the pathogenic mechanism of *V. alginolyticus* infection is urgently needed to guide the effective prevention and therapy for *V. alginolyticus* infection.

The major virulence factors of *V. alginolyticus* are quorum sensing (QS), extracellular proteases, motility, siderophore-dependent iron uptake systems, biofilm, adhesion, type III secretion systems (T3SS), and type VI secretion systems (T6SS) [15]. QS systems are intricately regulated as the global regulatory networks of gene expression in bacteria. In *V. alginolyticus*, a *V. harveyi*–like QS signaling system was characterized, which involved pivotal LuxO and LuxR regulators [16–21]. Multiple virulence factors and some other virulence-related genes were closely regulated by the QS system in *V. alginolyticus*. For example, in *V. alginolyticus*, motility and biofilm formation were proposed as important virulence-associated factors that could be regulated by QS [22–24]. AphA, another master regulator of QS, could also modulate the *in vivo* survival of *V. alginolyticus* in zebrafish [25]. In addition, QS was proved to control the activation of T6SS2 in *V. alginolyticus* [26,27]. Some studies also explored the role of other virulence factors in *V. alginolyticus*, but in general, most of the studies on *V. alginolyticus* in the last 20 years focused on virulence-related genes.

Bacterial noncoding (nc) RNAs are often small regulatory RNAs (<500 nt) [28]. To date, more than 100 ncRNAs have been identified in *Escherichia coli* [29]. Most chromosome-encoded ncRNAs are found to interact directly with their target mRNAs, thus influencing the translation initiation and/or mRNA stability [30]. A short complementary region of about 7–9 bp is commonly required for ncRNA–mRNA interaction [31]. Although the conventional RNA interference (i) pathway has not yet been identified in bacteria, bacterial ncRNAs have displayed several key roles in many biological processes by binding to the mRNA targets [32,33]. Bacterial ncRNAs regulate responses to environmental stresses [29], including translation control, RNA degradation, and RNA processing. They have been shown to regulate a wide variety of biological processes, including secretion, QS, stress responses, biofilm formation, adhesion, and virulence [34–36].

Despite detailed studies on bacterial ncRNAs, the functions of most of the bacterial ncRNAs have not been verified. Especially the role of ncRNAs in virulence regulation is still poorly understood. At present, only a very small number of ncRNAs are specifically elucidated in the regulation of virulence. For example, 4.5S RNA deletion mutation results in a significant reduction in the virulence of *Streptococcus pyogenes* [37]. RNA III has been shown to regulate the expression of multiple virulence genes in *S. pneumoniae* [36]. At present, the study of ncRNA in *Vibrio* focuses mainly on *V. cholerae* and *V. harveyi* [38]. Thus, determining the respective functions of bacterial ncRNAs remains a major challenge.

A previous study [28] first explored the ncRNAs and genes in *V. alginolyticus* cultured under normal and stress conditions, such as Cu^2+^, Pb^2+^, Hg^2+^, and low pH, using RNA-seq to gain a broad spectrum of expression of potential ncRNAs and genes associated with the bacterial adhesion. It also aimed to provide new clues for further understanding the mechanisms underlying the adhesion regulation in *V. alginolyticus*. Vibrio virulence regulatory RNA 1 (Vvrr1) is a new ncRNA found in *V. alginolyticus* using RNA-seq. Its expression level significantly increased in *V. alginolyticus* strains with impaired adhesion ability in the presence of heavy metal ions and under low-pH stress, indicating that Vvrr1 might be closely related to the adhesion ability of *V. alginolyticus*.

In this study, the target genes of Vvrr1 were fully screened and verified by constructing Vvrr1-overexpressing strains and using the proteome sequencing technology. *pykF* was found to be a chief target gene of Vvrr1 involved in virulence regulation. The molecular mechanism of Vvrr1 involved in the virulence regulation of *V. alginolyticus* was further revealed by exploring the mechanism of Vvrr1 regulating target genes and clarifying the influence of target genes on the virulence phenotype.

## Materials and methods

### Bacterial strains and culture conditions

Pathogenic *V. alginolyticus* (ND-01) was isolated from naturally infected *Pseudosciaena crocea* and previously identified as pathogenic by subsequent artificial infection. Bacteria were preserved in physiological saline with 10% glycerol at −80°C [1]. The ND-01 strain was routinely grown in Luria–Bertani (LB) medium at 28°C with shaking at 220 rpm. *E. coli* DH5α was obtained from TransGen Biotech (Beijing, China), which was grown in LB medium (37°C, 220 rpm).

### Construction of Vvrr1- and pykF-overexpressing strains

Competent bacterial cells were collected as mentioned earlier [39]. The study involved the pACYC184 vector, containing a short hairpin RNA (shRNA) comprising the entire sequence of Vvrr1, and a nontarget shRNA as control (5′-TTC TCC GAA CGT GTC ACG TTT-3′). The vector was transferred into SM10 using heat shock and then transferred via conjugation from SM10 to *V. alginolyticus*. Chloromycetin was used to screen positive clones [28]. The expression level of Vvrr1 was detected by quantitative real-time polymerase chain reaction (qRT-PCR).

The *pykF*-overexpressing strain was constructed with the pET28a vector. The full length of *pykF* was ligated to the linearized pET28a vector using T4 DNA ligase (New England Biolabs) based on the manufacturer’s recommendations. The vector was transferred into SM10 using heat shock and then transferred via conjugation from SM10 to *V. alginolyticus*. Kanamycin was used to screen the positive clones. Finally, the expression level of *pykF* was evaluated by qRT-PCR.

### Construction of Vvrr1 mutant strain

Based on the *V. alginolyticus* Vvrr1 gene sequence, primers with homologous arms were designed with Primer Premier 5.0 and synthesized (Table S1). The 5′ termini of the primers were homologous to the 35-bp upstream and downstream flanking regions of the Vvrr1 gene. The 3′ termini of the primers were homologous to the end of the tetracycline resistance gene. After PCR amplification, the targeting fragments (with tetracycline resistance) of Vvrr1 was constructed.

Plasmid pKD46 was transformed into *V. alginolyticus* and cultured to an OD_600_ = 0.3. After adding 30 mmol/L L-arabinose, the recombinant enzymes Exo, Bet, and Gam of pKD46 were fully expressed. The targeting fragments were then transformed into *V. alginolyticus* by electroporation. The positive clones were screened using tetracycline (10 μg/mL), and positive colonies were selected for PCR analysis and gene sequencing verification [40].

### Proteome analysis

Proteomics analysis was carried out with the iTRAQ technology (Applied Biosystems Incorporation). The protein was extracted from the Vvrr1-overexpressing strain and ND-01 (*n* = 3). The samples were labeled with iTRAQ reagents as described by Ye et al. [41]. LC-MS/MS analyses were carried out using an LTQ Orbitrap Velos (Thermo Fisher Scientific) with an Agilent 1100 binary high-pressure liquid chromatography pump (Agilent Technologies) and a FAMOS autosampler (LC Packings). Scaffold Q+ was used for protein identification and quantification with iTRAQ. Further quantitative data analyses were qualified on unique proteins with at least two unique peptides and a false discovery rate (FDR) <0.01. The fold changes in protein abundance were defined as the median ratios of all significantly matched spectra with tag signals [41]. The peak identification, establishment of a reference database, and peptide and protein identification were conducted. GO functional classification annotation and KEGG pathway annotation were performed on all identified proteins, and differential proteins were screened and analyzed according to the differences in multiples and *P* values (*P* value < 0.05). The result of iTRAQ has been deposited to the ProteomeXchange Consortium via the PRIDE partner repository with the dataset identifier PXD014375.

### Construction of gene-silenced strain

The *pykF-*, *ndk-*, *eno-*, *sdhB-*, *glpF-*, and *cysH-*silenced strain were constructed as previously described [42]. The shRNA sequences targeting *pykF*, *ndk*, *eno*, *sdhB*, *glpF*, and *cysH* were designed and synthesized by Shanghai Generay Biotech Co., Ltd. (Shanghai, China) (Table S1). After linearizing pACYC184 vectors with the restriction enzymes *Bam*HI and *Sph*I (New England Biolabs, USA), the oligonucleotides were annealed and ligated to the linearized pACYC184 vectors using T4 DNA ligase (New England Biolabs) following the manufacturer’s recommendations. The recombinant pACYC184 vectors were transferred into SM10 using heat shock and then transferred via conjugation from SM10 to *V. alginolyticus*. Chloromycetin was used to screen the positive clones. Finally, the expression levels of *pykF*, *ndk*, *eno*, *sdhB*, *glpF*, and *cysH* of each RNAi strain were detected using qRT-PCR.

### GFP reporter gene analysis

The full-length sequence of *pykF* with or without the interaction sequence against Vvrr1 was synthesized by Shanghai Generay Biotech Co., Ltd. After linearizing pET28a-GFP vectors and *pykF* or *pykF*ΔIS gene with the restriction enzymes *Kpn*I and *Hind*III (TransGen Biotech), the *pykF* or *pykF*ΔIS gene was ligated to the linearized pET28a-GFP vectors using T4 DNA ligase (New England Biolabs) following the manufacturer’s recommendations. The recombinant pET28a-GFP-*pykF* or pET28a-GFP-*pykF*ΔIS vectors were transferred into the competent *E. coli* DH5α cells using heat shock. Kanamycin was used to screen the positive clones. Then, the pACYC184 vector containing the entire ncRNA sequence of Vvrr1 with or without the interaction sequence against *pykF* was transferred into the positive clones by electrotransformation. Single bacterial colonies were screened with kanamycin and chloromycetin. The fluorescence was observed using a fluorescence microscope and imaged with a digital video camera.

### Flow cytometric analysis

The flow cytometric analysis was performed using a method modified from another study [43]. Single bacterial colonies were inoculated in 2 mL of LB medium containing kanamycin and chloromycetin and grown at 37°C and 220 rpm for 12 h. A culture volume corresponding to one OD_600_ per milliliter was mixed with 1 mL of phosphate-buffered saline (PBS) (pH 7.4) containing 4% paraformaldehyde and centrifuged for 2 min at 7500 *g*. The pellets were resuspended in 1 mL of 1× PBS. For flow cytometry analysis, 1/1000 dilutions in 1× PBS were prepared and measured on a CyFlow Space machine. Biological triplicates were prepared for every sample.

### RNA extraction and reverse transcription

TRIzol reagent (Invitrogen, USA) was used for total RNA extraction from bacterial cells following the manufacturer’s protocol. Reverse transcription was carried out with a Reverse Aid Mu-MLV cDNA synthesis kit (TransGen Biotech) from 2.0 mg total RNA following the manufacturer’s protocol [44].

### qRT-PCR

qRT-PCR was carried out using a QuantStudio 6 Flex real-time PCR system (Life Technologies). All primer sequences are provided in Table S1. The expression levels of bacterial genes were normalized using *gyrB* rRNA. The 2^−ΔΔCt^ method was used to calculate the relative level of gene expression [45].

### Northern blot analysis

For the Northern blot analysis of Vvrr1, 20 mg total RNA was loaded into each lane, and ribosomal RNA bands were visualized using ethidium bromide staining. The 5′-biotin-labeled oligonucleotides used as probes are listed in Table S1. The probes were detected on the membranes using the streptavidin–Alexa Fluor 680 conjugate (Invitrogen, CA, USA) according to the manufacturer’s protocols.

### In vitro adhesion assay

The bacterial adhesion was assayed, as described by Huang et al. [28]. A total of 20 µL of the mucus was evenly spread onto a glass slide area of 22 × 22 mm^2^. After fixing with methanol for 30 min, 200 µL of the bacterial suspension (10^8^ CFU/mL) was added onto the mucus-coated glass slides. After incubating at 28°C for 2 h in a humidified chamber, the slides were washed five times with PBS to remove nonadherent bacterial cells. Finally, the adhering bacterial cells were fixed with 4% methanol for 30 min, dyed with crystal violet for 3 min, and observed under a microscope (×1000). The average number of bacteria adhering to a field of view of the glass surface was then determined. For each assay, 20 fields of view were counted, and the average value was calculated.

### Growth curve assay

*V. alginolyticus* strains were overnight grown at 28°C in LB and adjusted to OD_600 nm _= 0.05. Then, 50 μL of the bacterial culture was mixed with 150 μL of LB per well of a 96-well plate and then incubated at 28°C. The concentration of the bacterial solution was measured every hour for a total of 24 h, and the values of OD_600 nm_ were recorded. From the OD_600 nm_ data, the growth curves were plotted to compare the wild-type, overexpressing strain and RNAi strain [46].

### Hemolysis assay

Hemolysis assays were carried out as described by Tsou and Zhu [47]. Sheep blood (Ping Rui Biotechnology Co. Ltd. Beijing, China) was washed three times with PBS. A total of 5 μL of the washed sheep blood was mixed with 245 μL of culture supernatants and incubated at 37°C for 1 h with shaking (150 rpm). After incubation, the samples were centrifuged, and the released hemoglobin content was measured using OD_540 nm_. The percentage of total hemolysis was calculated by comparing the OD_540 nm_ of the samples with positive (ddH_2_O) and negative controls (PBS). Three independent biological replicates were performed for each data point.

### Biofilm assay

The biofilm assay for *V. alginolyticus* was performed as described by Luo et al. [39]. *V. alginolyticus* strains were overnight grown at 28°C in LB and then adjusted to OD_600 nm _= 0.2. A total of 50 μL of the bacterial culture was mixed with 150 μL of LB per well of a 96-well plate and then incubated at 28°C for 24 h. Then, it was washed twice with sterile PBS, incubated with 175 μL of crystal violet (0.1%) for 15 min, washed with sterile PBS, and air-dried. The stained biofilm was solubilized with 200 μL of 33% acetic acid and then quantitated by measuring OD_590 nm_. Six independent biological replicates were performed for each data point.

### Soft agar plate motility assay

The soft agar method was used to assay the motility of *V. alginolyticus* strains. Fresh overnight cultures were diluted to an OD_600 nm_ of 0.3 in LB. The cell suspension (1 μL/drop) was dropped onto the center of semisolid agar plates (LB broth + 0.3% agar), and the plates were allowed to incubate overnight at 28°C before measuring the colony diameters. Three independent biological replicates were performed per group.

### Artificial infection and sampling

*Epinephelus coioides* (average weight 50.0 ± 2.0 g) fish were obtained from Zhangzhou (Fujian, China) and acclimatized at 18°C for 1 week under specific pathogen-free laboratory conditions. Fish were tested to be healthy using sera agglutination and bacteriological recovery tests. *E. coioides* fish were divided into several groups (*n* = 10 in each group; triplicates were used for each experiment) and grown in 500-L tanks with constant aeration and a flow-through setup.

For the survival assays, the weight-matched *E. coioides* fish were intrapleurally injected with 10^3^ CFU/g of *V. alginolyticus* (wild-type, overexpressing strain or RNAi strain). *E. coioides* intrapleurally injected with PBS were used as negative control. The water temperature during infection was 18 ± 1°C. The daily mortality of infected *E. coioides* was recorded [48].

### Statistical analysis

The results were reported as mean ± SD. The data were statistically analyzed with one-way ANOVA followed by Dunnett’s multiple-comparison tests via SPSS 13.0 software. A *P* value <0.05 was used to indicate a significant difference.

### Ethics statement

All animal experiments were conducted strictly based on the recommendations in the “Guide for the Care and Use of Laboratory Animals” set by the National Institutes of Health. The animal protocols were approved by the Animal Ethics Committee of Jimei University (Acceptance No. JMULAC201159).

## Results

### Proteomic analysis of Vvrr1-overexpressing strain and ND-01 strain

**Figure 1:**
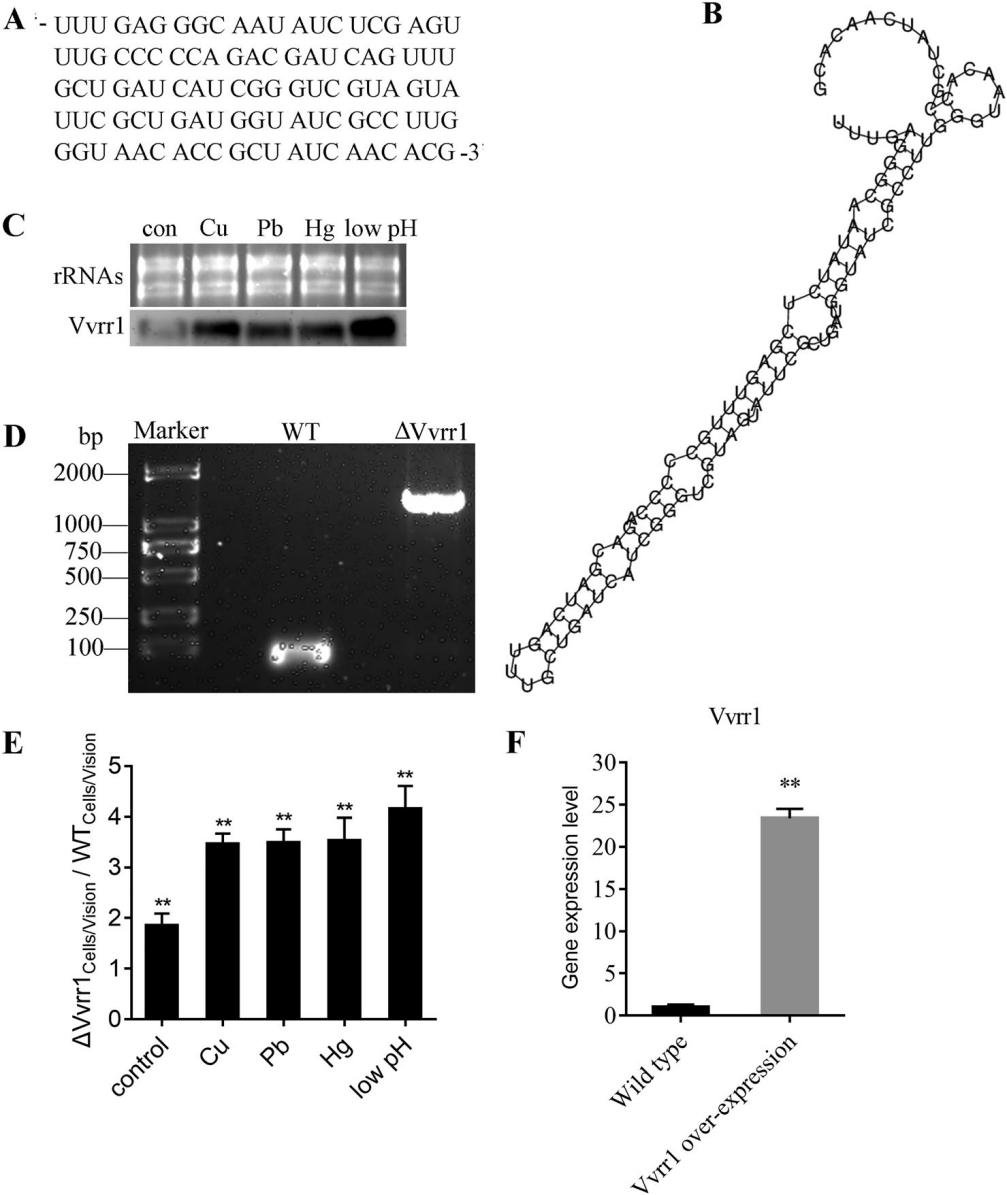
. (A) Sequence of Vvrr1. (B) Secondary-structural prediction for Vvrr1 identified in *V. alginolyticus*. (C) Expression level of Vvrr1 in *V. alginolyticus* under stresses detected by the Northern blot analysis (lower panel). The ribosomal (r) RNA bands stained with ethidium bromide (upper panel) were used as loading controls. (D) Construction and confirmation of the knockout mutant strain ΔVvrr1. WT: Amplification of wild-type using primers Vvrr1_mut_-F-R; ΔVvrr1: amplification of ΔVvrr1 using primers Vvrr1_mut_-F-R. (E) Comparison of adhesion ability in wild-type and ΔVvrr1 strains. Data are presented as mean ± SD. Three independent biological replicates were performed per group. *P < 0.05, **P < 0.01. (F) Expression level of Vvrr1 in the wild-type and Vvrr1-overexpressing strains identified by qRT-PCR. Data are shown as mean ± SD from three independent biological replicates. **P < 0.01.

Vvrr1 was an ncRNA comprising 105 nucleotides identified using RNA-seq and predicted to be responsible for the impaired adhesion ability under environmental stress (Figure 1A and 1B). In the present study, the existence of Vvrr1 was successfully validated by Northern blot analysis, revealing a ∼105-nt RNA expressed from the positive strand in *V. alginolyticus.* Meanwhile, the Northern blot analysis showed that the expression level of Vvrr1 significantly increased under stresses, including Cu^2+^, Pb^2+^, Hg^2+^, and low pH, which was consistent with the results of previous RNA-seq (Figure 1C). To further document the necessity of Vvrr1 for adhesion regulation under environmental stress, the Vvrr1 knockout strain was constructed (Figure 1D) and its adhesion ability under different stresses was tested. The results showed that the decline of bacterial adhesion was rescued when Vvrr1 was knocked out (Figure 1E).

**Figure 2.**
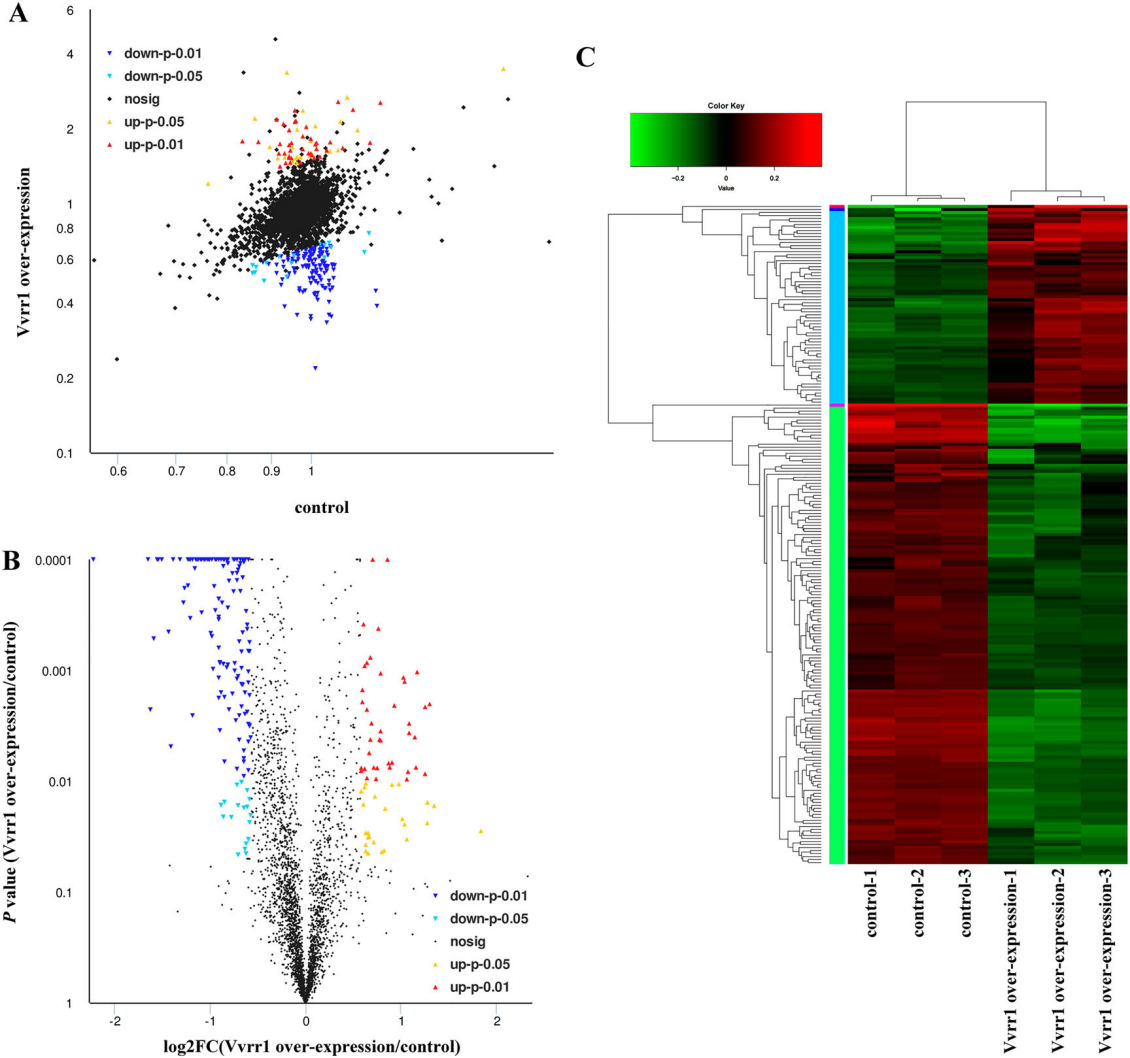
(A) Differential protein visualization scatter map. The horizontal and vertical coordinates represent the protein expressions in the two groups. (B) Volcano plot of differential proteins. The abscissa is the multiple change value of protein difference between the two groups, and the ordinate is the statistical *t* test *P* value of the protein expression difference. Each point in the figure represents a specific protein: the yellow point represents the protein significantly upregulated at *P* < 0.05, the red point represents the protein significantly upregulated at *P* < 0.01, the light blue point represents the protein significantly downregulated at *P* < 0.01, the blue point represents the protein significantly downregulated at *P* < 0.01, and the black point represents the nonsignificantly differentially differentiated protein. (C) Hierarchical clustering of differentially expressed proteins. Green and red indicate decreased and increased expression, respectively.

**Figure 3.**
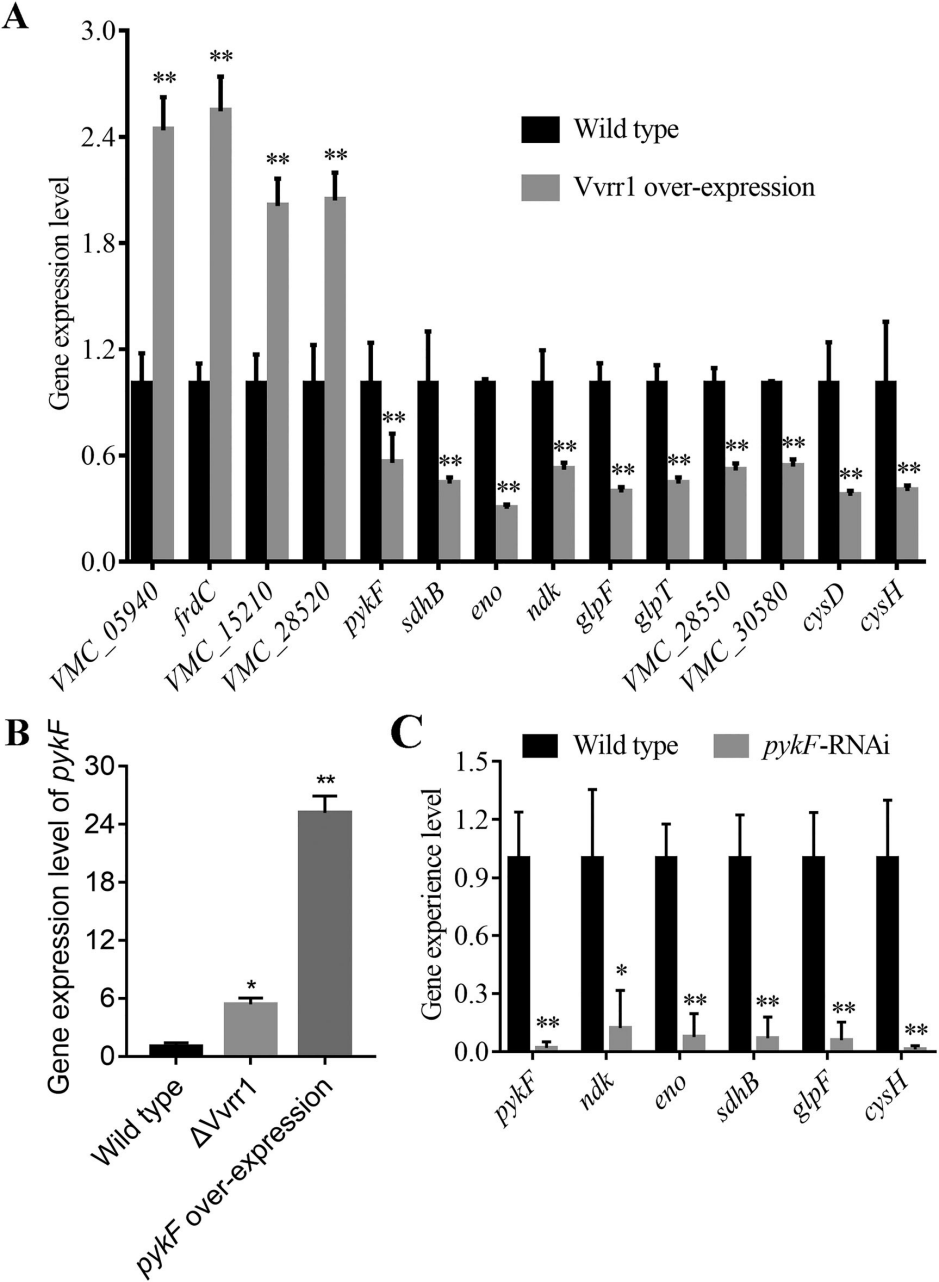
qRT-PCR analysis of the expression of virulence genes in wild-type, Vvrr1-overexpressing (A), ΔVvrr1, *pykF*-overexpressing (B) and *pykF*-RNAi (C) strains. Data are presented as mean ± S.D. (n = 3). *P < 0.05, **P < 0.01.

To investigate the function of Vvrr1, a Vvrr1-overexpressing strain was constructed, which significantly increased the expression level of Vvrr1 by 23.4-fold (± 1.1) (Figure 1F). The quantitative proteomic analysis was used to compare the expression levels of proteins in the Vvrr1-overexpressing strain and ND-01 strain, and significant changes in protein expression levels were observed (Figure 2). The results revealed 3062 proteins in the species, of which 219 were differentially expressed (66 upregulated and 153 downregulated). A total of 26 and 18 proteins were significantly upregulated and downregulated, respectively (*P* < 0.05), while 40 and 135 proteins were extremely significantly upregulated and downregulated, respectively (*P* < 0.01) (Table S2). The qRT-PCR was performed on randomly selected genes to validate the results of sequencing. The results of qRT-PCR matched the sequencing results (Figure 3A). These data reinforced the reliability of the sequencing data.

The functions of the differentially expressed proteins were analyzed according to GO, and the upregulated and downregulated proteins mapped to every term were counted. This analysis categorized 219 differentially expressed proteins into 27 secondary classifications (data not shown). The analysis of GO categories showed that most of the differentially expressed proteins were involved in the following functional categories: metabolic process, cellular process, membrane, membrane part, cell part, binding, and catalytic activity. The number of proteins involved in catalytic activity and metabolic processes was the largest. According to this analysis, 66 upregulated and 153 downregulated proteins were categorized into 25 and 20 enriched functional groups, respectively (data not shown). The enrichment ratio of the upregulated and downregulated proteins involved in carbon utilization and biological adhesion was the highest. The differentially expressed proteins were extremely significantly enriched in localization, membrane, membrane part, transporter activity, and catalytic activity (Data not shown).

Next, the pathway analysis of differentially expressed proteins was performed on the basis of the latest version of the KEGG database (https://www.kegg.jp/), and the differentially expressed proteins were assigned to 60 KEGG pathways (data not shown). The results revealed that the protein enrichment ratio in the TCA cycle and the sulfur metabolism pathway was the highest, and the significance was the strongest (*P* < 0.001).

To obtain the interaction relationship between differentially expressed proteins, the STRING database (https://string-db.org/) was used for prediction and analysis. Figure S1 shows that the protein PykF acted as a hub protein, having maximum connectivity. Further, *eno* and *ndk* were directly related to *pykF*, and the correlation was strong. *sdhB, glpF*, and *cysH* had an indirect and relatively weak correlation with *pykF* (Figure S1). To validate the regulatory effect of Vvrr1 on the hub protein PykF encoding gene, the expression level of *pykF* was detected in the Vvrr1 knockout strain. The results showed that when Vvrr1 was knocked out, the expression level of *pykF* increased, which was consistent with the results of qRT-PCR in the Vvrr1-overexpressing strain and indicated a negative regulatory effect of Vvrr1 on *pykF* (Figure 3B). To determine the correlation between the aforementioned five genes and *pykF,* the *pykF*-silencing strain was constructed and the silencing effect and the expression level of other genes in the silencing strain were further verified with qRT-PCR. This revealed that the expression levels of the five genes significantly reduced when *pykF* was silenced (Figure 3C), indicating a positive regulatory effect of *pykF* on them.

IntaRNA was used to predict the interactions between Vvrr1 and the mRNA of significantly changed proteins. The interaction network diagram of Vvrr1 with its potential target genes was predicted with the STRING database (Figure S2). When Vvrr1 was overexpressed, the protein expression levels of *sdhA*, *relA*, *msrA*, *pykF*, and *hutU* decreased, and a number of other proteins connected with them. Figures S1 and S2 show that *pykF* was a common gene in the two network diagrams. This implied that *pykF* was not only regulated by Vvrr1, but also a hub in the protein interaction regulation network, which might have biological importance. Therefore, it was selected as the potential key target gene of Vvrr1 for further study.

### Regulatory effect of Vvrr1 and its target genes on V. alginolyticus virulence

**Figure 4.**
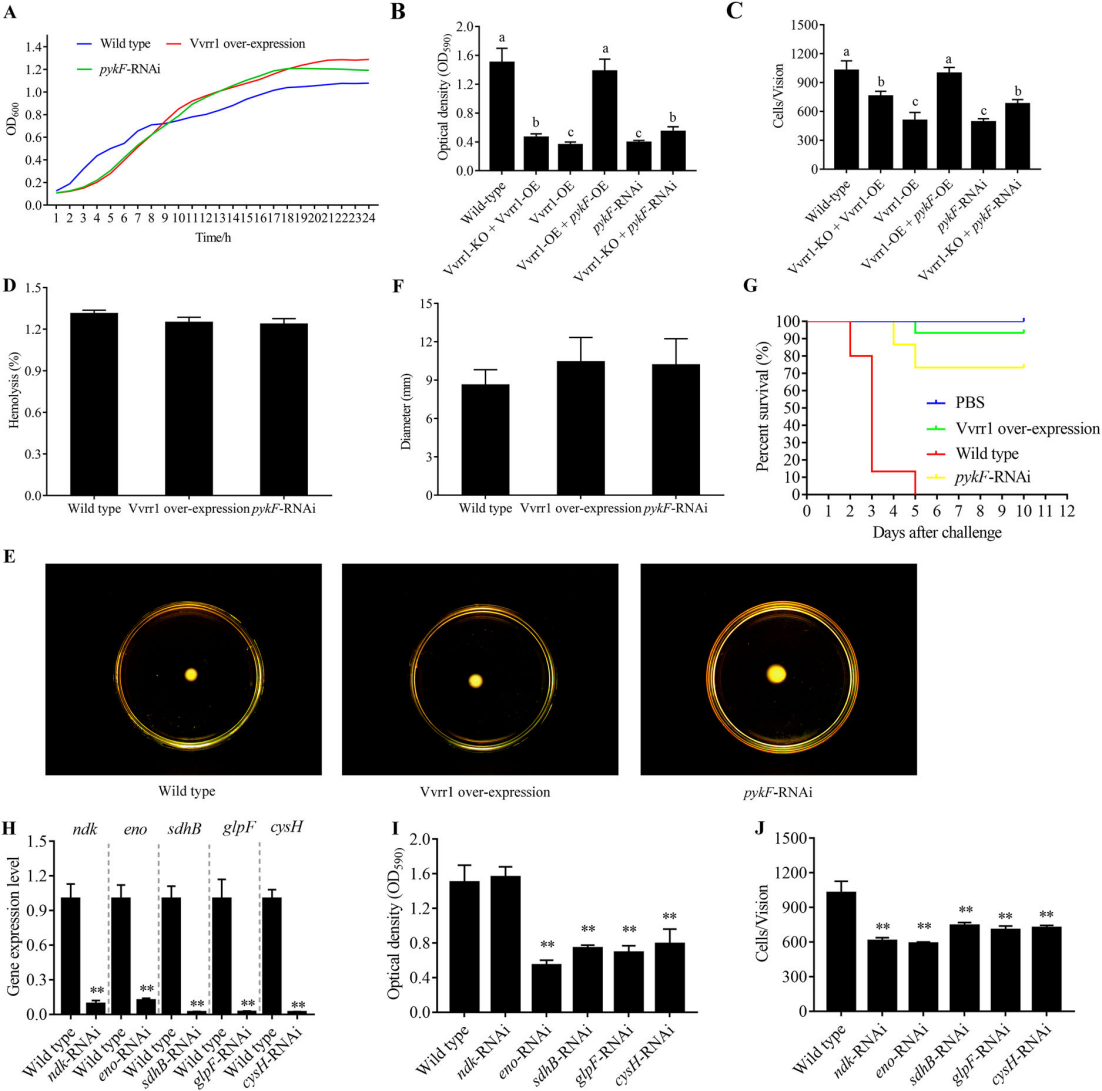
The growth curve (A), biofilm formation (B), adhesion ability (C), hemolysis (D), motility (E and F) and survival rate (G) were measured in wild-type, Vvrr1-overexpressing, *pykF*-RNAi, Vvrr1 knockout + Vvrr1-overexpressing, Vvrr1-overexpressing + *pykF*-overexpressing, and Vvrr1 knockout + *pykF*-RNAi strains. The contribution of *ndk*, *eno*, *sdhB*, *glpF* and *cysH* to the biofilm formation (I) and the adhesion ability (J) were compared after gene silencing (H). Data are presented as mean ± SD. Three independent biological replicates were performed per group. *P < 0.05, **P < 0.01.

The growth curves of wild-type, Vvrr1-overexpressing, and *pykF*-RNAi strains were evaluated. The results showed that the growth rate of Vvrr1-overexpressing and *pykF*-RNAi strains increased slightly compared with the wild-type strain (Figure 4A). It indicated that ncRNA Vvrr1 and *pykF* gene were probably involved in the growth of *V. alginolyticus*. The comparison of biofilm formation and adhesion among wild-type, Vvrr1-overexpressing, *pykF*-RNAi, Vvrr1 knockout + Vvrr1-overexpressing, Vvrr1-overexpressing + *pykF*-overexpressing, and Vvrr1 knockout + *pykF*-RNAi strains showed that Vvrr1 and *pykF* played a role in the biofilm development and adhesion of *V. alginolyticus* (Figure 4B and 4C). However, the Vvrr1-overexpressing and *pykF*-RNAi strains showed no significant difference in hemolysis and motility compared with the wild-type strain (Figure 4D–F). These results indicated that Vvrr1 and *pykF* gene contributed to the multiple steps of the pathogenesis of *V. alginolyticus*. The artificial infection also displayed a significantly reduced virulence of the Vvrr1-overexpressing strain and the *pykF*-RNAi strain (Figure 4G). Meanwhile, the contribution of *ndk*, *eno*, *sdhB*, *glpF*, and *cysH* to the adhesion ability and biofilm formation was detected after their gene silencing (Figure 4H–4J), suggesting that *eno*, *sdhB*, *glpF*, and *cysH* were involved in the regulation of adhesion ability and biofilm formation, while *ndk* was involved only in the regulation of adhesion ability.

### Pykf was a target gene of Vvrr1 regulating virulence

**Figure 5.**
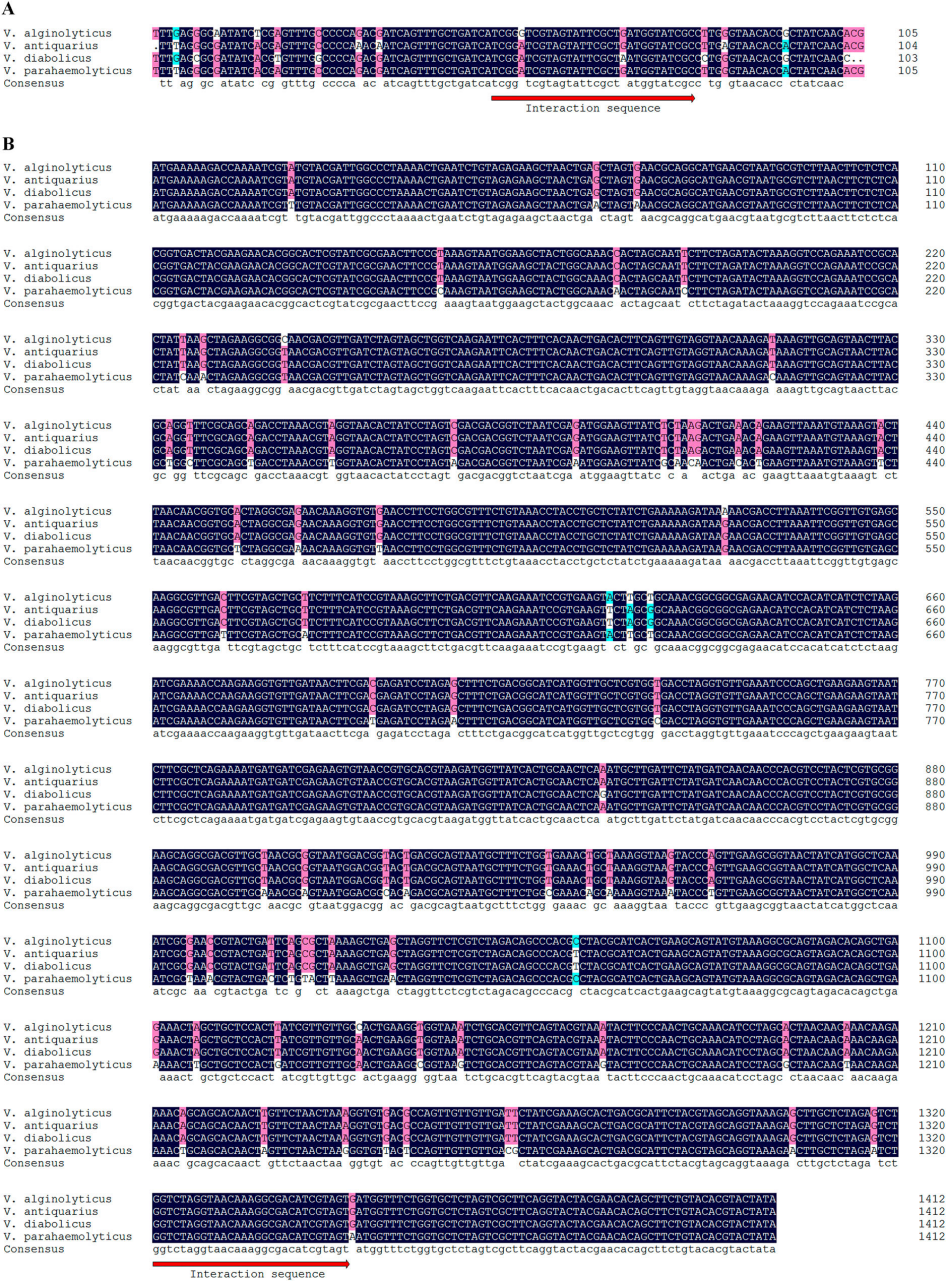
Alignment of Vvrr1 and *pykF* identified in *V. alginolyticus*, *V. parahaemolyticus*, *V. antiquarius*, and *V. diabolicus*. The putative Vvrr1-pykF interaction sequence is indicated by the red arrow. The numbering of residues follows the sequence.

The DNA sequence for the Vvrr1 in *V. alginolyticus* was BLAST against the genome of other Vibrios on the basis of the latest version of the BLASTN, indicating that the Vvrr1 of *V. alginolyticus* shared 95%, 92%, and 92% identities with the homologous genes present in *V. parahaemolyticus*, *V. antiquarius*, and *V. diabolicus*, respectively. Furthermore, the interaction sequence against *pykF* in Vvrr1 was identified by IntaRNA and highlighted as having ∼100% sequence conservation across several Vibrios (Figure 5). Thus, the alignment of DNA sequences for *pykF* in *V. alginolyticus*, *V. parahaemolyticus*, *V. antiquarius*, and *V. diabolicus* was also carried out, implying that the *pykF* of *V. alginolyticus* shared 95%, 99%, and 99% identities with the homologous genes present in *V. parahaemolyticus*, *V. antiquarius*, and *V. diabolicus*, respectively. Meanwhile, the interaction sequence against Vvrr1 in *pykF* was also identified by IntaRNA and highlighted as having ∼100% sequence conservation across several Vibrios (Figure 5). The deep conservation of these two interaction sequences throughout Vibrios indicated their biological importance.

**Figure 6.**
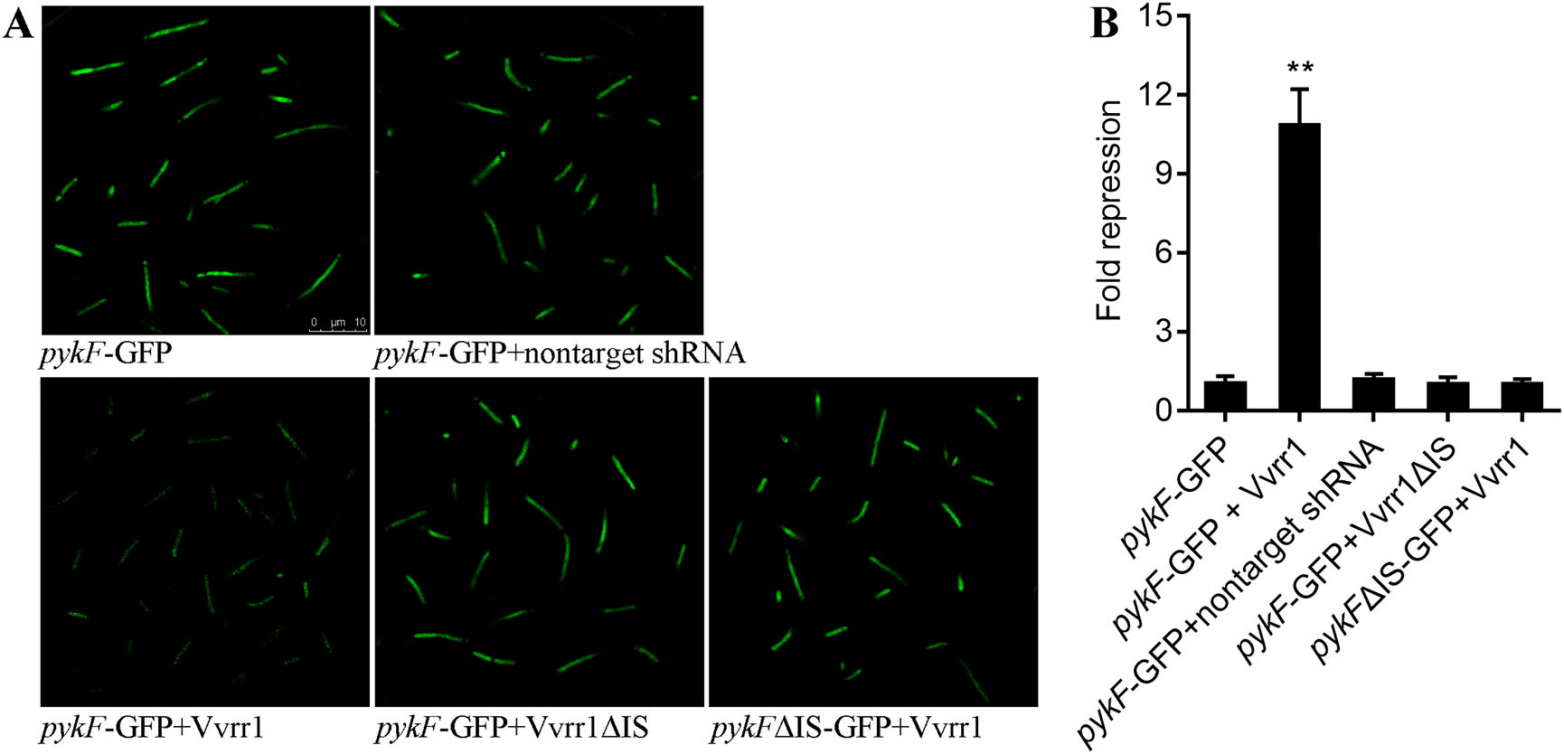
GFP reporter gene analysis revealed interactions between the ncRNA Vvrr1 and the mRNA of *pykF* gene. (A) Fluorescence intensities of *E. coli* containing *pykF*-GFP, *pykF*-GFP + nontarget shRNA, *pykF*-GFP + Vvrr1, *pykF*-GFP + Vvrr1ΔIS, and *pykF*ΔIS-GFP + Vvrr1 under fluorescence microscope. GFP fluorescence was excited at 460 nm, and light emission was recorded using a 510 nm filter. (B) Flow cytometric analysis of target regulation. Fold repression is depicted on the y-axis. Data are presented as the mean ± SD (n = 3, **P < 0.01).

To validate the results of the aforementioned analysis and further document the specific Vvrr1–*pykF* mRNA interactions, GFP reporter gene analysis was performed in *E. coli*. *E. coli* containing *pykF*-GFP plasmids displayed green fluorescence under the fluorescence microscope, while the fluorescence intensities were significantly repressed by vectors containing the entire ncRNA sequences of Vvrr1 (Figure 6A). The results of the flow cytometric analysis also showed that Vvrr1 was sufficient for the repression of *pykF* by 10.82-fold (Figure 6B). However, the absence of Vvrr1–*pykF* interaction sites caused a reduction of GFP translation repression in both genetic backgrounds, suggesting that both interaction sequences were involved in Vvrr1–*pykF* binding (Figure 6). These results reinforced the prediction of IntaRNA and indicated the interactions between the ncRNA Vvrr1 and the mRNA of *pykF*. Therefore, it was hypothesized that the regulatory mechanism of Vvrr1 against *pykF* played an important role in the pathogenicity of *V. alginolyticus*.

## Discussion

The pathogenic process of *V. alginolyticus* includes adhesion, invasion, proliferation *in vivo*, and production of toxins. The pathologic effect is caused by cell and tissue damage induced by *V. alginolyticus* during its invasion and proliferation, as well as its metabolites (toxins) interfering with and destroying the local metabolism or function of the partial or whole host body [49]. The adhesion to the host body or cell is a prerequisite for the pathogenic bacteria to cause disease. Therefore, effective blocking of the adhesion of pathogenic bacteria to the host is of great significance in the prevention and treatment of diseases [50].

Previous studies involved the transcriptomic analysis of the ncRNA of *V. alginolyticus* strains under different stress conditions [51], revealing that the expression level of Vvrr1, an un-reported ncRNA, significantly increased in the strains of *V. alginolyticus* with impaired adhesion ability under the stress of Cu^2+^, Pb^2+^, Hg^2+^, and low pH. This indicated that Vvrr1 might be closely related to the adhesion ability of *V. alginolyticus*. In the present study, the proteomics analysis showed that when Vvrr1 was overexpressed, only significantly downregulated proteins existed in the biological adhesion GO enrichment, and their enrichment rate was the highest in all biological processes. At the same time, the KEGG pathway analysis of downregulated proteins showed that the protein enrichment rate of the TCA cycle and sulfur metabolism was the highest and the most significant, while the TCA cycle was confirmed to have an important regulatory effect on the adhesion process of *V. alginolyticus* [52]. These findings also indicated that Vvrr1 participated in the adhesion of *V. alginolyticus*. When Vvrr1 was overexpressed, the adhesion ability of *V. alginolyticus* significantly decreased, further indicating that Vvrr1 played a crucial role in the adhesion process.

**Figure 7.**
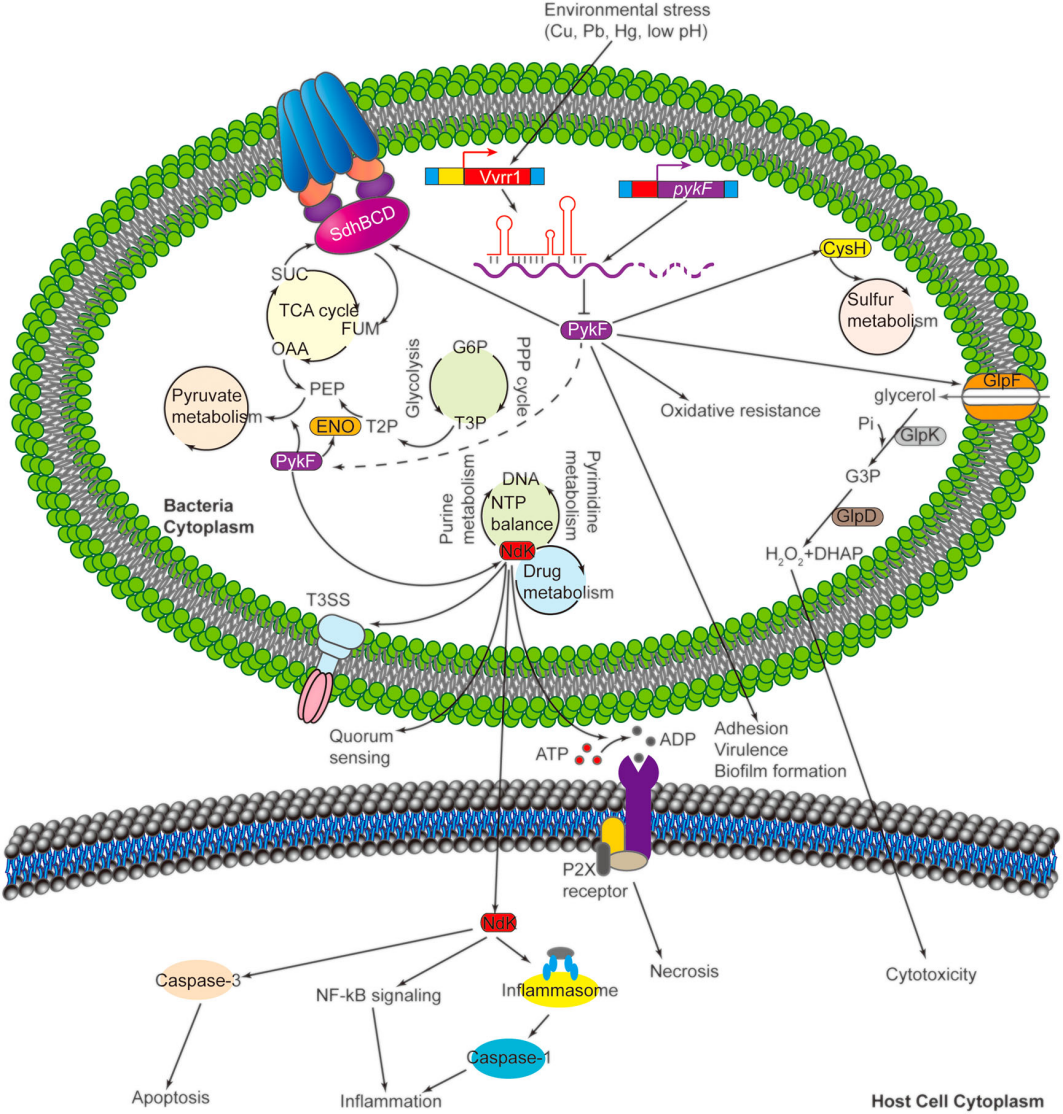
Working model of Vvrr1-*pykF* axis control of metabolic and virulence related processes in *V. alginolyticus*. Various stresses including Cu^2+^, Pb^2+^, Hg^2+^ and low pH modulate the level of Vvrr1. Vvrr1 binds to *pykF* mRNA and reduce its translation, thus affecting the expression of *ndk*, *eno*, *sdhB*, *glpF* and *cysH.* Arrowed and bar-ended lines indicate activation and repression, respectively.

IntaRNA was used to predict the target genes of Vvrr1 by sequence analysis in the present study. Vvrr1 had multiple potential target genes closely related to bacterial virulence. It negatively correlated with the expression levels of these genes, such as *pykF*. Further, *pykF*, a key metabolic gene that encodes pyruvate kinases associated with carbon metabolism, is widely present in many intracellular bacteria. In recent years, the relationship between the carbon metabolism of intracellular bacteria and their virulence has become a research hotspot. Many intracellular bacteria, such as *Listeria monocytogenes*, *Shigella*, and *Mycobacterium tuberculosis*, can adapt to the harsh living environment by regulating their own metabolism. In addition, *Brucella* strains lacking the pyruvate kinase gene *pykF* were found to have significantly reduced viability in host macrophages [53–55]. Meanwhile, the *pykF*-centric network diagram in the present study demonstrated that *ndk* was a manipulation of bacterial virulence and adaptive responses of the pleiotropic effector. Studies have revealed that *ndk* secreted from bacteria is important in modulating virulence-associated phenotypes, including QS regulation, type III secretion system activation, and virulence factor production (Figure 7). Moreover, after infection, *ndk* released from bacteria are involved in regulating host defense activities, such as cell apoptosis, phagocytosis, and inflammatory responses [56]. In addition, *ndk* is a critical novel host-responsive gene required for coordinating *P. aeruginosa* virulence upon acute infection, and its mutant exhibits enhanced cytotoxicity and host pathogenicity by increasing the level of T3SS proteins [57]. The interaction network diagram of Vvrr1 with its potential target genes shows some of the target genes with their virulence function. For example, *relA* has been proved to enhance the adhesion of *E. coli* to host cells [58]. *katG* can increase the survival and escape rates of *Aeromonas hydrophila* in fish macrophages [59]. These observations indicated that Vvrr1 might play an important role in the virulence gene regulatory network of *V. alginolyticus*.

ncRNA can block protein translation by binding to the mRNA of the target gene, or regulate the gene after transcription by promoting the degradation of mRNA, both leading to the decrease in the protein expression level encoded by the target gene. *pykF*, the hub gene of the interaction network diagram of differentially expressed proteins, was chosen for further research. The results of this study strongly suggested that Vvrr1 negatively affected *pykF* translation through direct binding to *pykF* mRNA at 1320–1350 bp located on mRNA. However, a reduction of *pykF* mRNA was also observed after Vvrr1 overexpression. Thus, Vvrr1 might also promote the degradation of *pykF* mRNA. However, further investigation is still required to confirm this.

In the present study, Vvrr1 and *pykF* were found to be negatively and positively related to adhesion, biofilm production, and virulence in *V. alginolyticus*, while *pykF* was negatively regulated by Vvrr1. Meanwhile, the *pykF*-centric network diagram in the present study showed that multiple downstream virulence genes, including *ndk*, *eno*, *sdhB*, *glpF*, and *cysH*, were positively regulated by *pykF*. Moreover, *eno*, *sdhB*, *glpF*, and *cysH* were involved in the regulation of adhesion ability and biofilm formation, while *ndk* was involved only in the regulation of adhesion ability, consistent with reports on other bacterial strains. For example, *ndk* (nucleoside-diphosphate kinase encoding gene) and *eno* (enolase encoding gene) have been implicated in the adhesion of *Burkholderia pseudomallei* [60]. The *Lactobacillus plantarum* Eno is involved in biofilm development [61]. *sdhB* (succinate dehydrogenase iron-sulfur subunit encoding gene) is found to be necessary for the adhesion and biofilm formation in *Staphylococcus aureus* [62]. *glpF* (glycerol uptake facilitator protein-encoding gene) participates in *L. monocytogenes* biofilm formation at the air–liquid interface [63] and adhesion to Caco-2 cells [64]. *cysH* (phosphoadenosine phosphosulfate reductase encoding gene) is related to the regulation of adhesion and biofilm formation in *E. coli* [65]. These findings confirmed that the Vvrr1–*pykF* axis was associated with the regulation of *V. alginolyticus* pathogenicity (Figure 7). Interestingly, according to the KEGG database, *pykF*, *ndk*, *eno*, *sdhB*, *glpF*, and *cysH* were reported to be closely related to the microbial metabolism in diverse environments, indicating the importance of the Vvrr1–*pykF* axis in the adaptation and response to environmental stresses (Figure 7).

This study was novel in revealing the molecular mechanism of Vvrr1 involvement in the virulence regulation of *V. alginolyticus* by exploring the mechanism of the Vvrr1 regulation of target genes. The findings might help understand the virulence regulation network of *V. alginolyticus* and provide the reference value for the functional identification of *V. alginolyticus* and other bacterial ncRNA.

## Supplementary Material

Supplemental Material
