# Supplementary material for: Mechanisms underlying the virulence regulation of new Vibrio alginolyticus ncRNA Vvrr1 with a comparative proteomic analysis
Source: Emerg Microbes Infect. 2019 Nov 12;8(1):1604–18. doi: 10.1080/22221751.2019.1687261 (PMC6853220; doi:10.1080/22221751.2019.1687261)
Supplement: Supplemental Material [file TEMI_A_1687261_SM9120.zip › Supplementary Material/Table S1.docx]

**Table S1. Sequences of primers used in the study**

|  | **shRNA sequence for stable gene silence** | |
| --- | --- | --- |
| *pykF*-RNAi | F:5'-GATCCGCACTCGTATCGCGAACTTTTCAAGAGAAAGTTCGCGATACGAGTGCTTTTTTGCATG-3'  R:5'-GCGTGAGCATAGCGCTTGAAAAGTTCTCTTTCAAGCGCTATGCTCACGAAAAAAC-3' | |
| *ndk*-RNAi | F:5'-GATCCGCTTTAGAGCGAAACCTAATTTTCAAGAGAAATTAGGTTTCGCTCTAAAGCTTTTTTGCATG-3'  R:5'-GCGAAATCTCGCTTTGGATTAAAAGTTCTCTTTAATCCAAAGCGAGATTTCGAAAAAAC-3' | |
| *eno*-RNAi | F:5'-GATCCGCTGCAATCGACGCAGTAATGTTCAAGAGACATTACTGCGTCGATTGCAGCTTTTTTGCATG-3'  R:5'-GCGACGTTAGCTGCGTCATTACAAGTTCTCTGTAATGACGCAGCTAACGTCGAAAAAAC-3' | |
| *sdhB*-RNAi | F:5'-GATCCGAGCAAGCCCGTGAAAGAAAGTTCAAGAGACTTTCTTTCACGGGCTTGCTCTTTTTTGCATG-3'  R:5'-GCTCGTTCGGGCACTTTCTTTCAAGTTCTCTGAAAGAAAGTGCCCGAACGAGAAAAAAC-3' | |
| *glpF*-RNAi | F:5'-GATCC GCCATCGCTATTTACTGTACG TTCAAGAGA CGTACAGTAAATAGCGATGGC TTTTTTGCATG-3'  R:5'-G CGGTAGCGATAAATGACATGC AAGTTCTCT GCATGTCATTTATCGCTACCG AAAAAAC-3' | |
| *cysH*-RNAi | F:5'-GATCCGCTGGAGCTCACAGAGTTACTTTCAAGAGAAGTAACTCTGTGAGCTCCAGCTTTTTTGCATG-3'  R:5'-GCGACCTCGAGTGTCTCAATGAAAGTTCTCTTCATTGAGACACTCGAGGTCGAAAAAAC-3' | |
|  | **Primers for the construction of the mutants** | |
| Vvrr1mut-F | 5'-TTTGAGGGCAATATCTCGAGTTTGCCCCCAGACGAGTGAAACCCAACATA-3' | |
| Vvrr1mut-R | 5'-CGTGTTGATAGCGGTGTTACCCAAGGCGATACCATTCAGCGATCGGCTCG-3' | |
|  | **Probes for northern blot analysis** | |
| Vvrr1 | 5'-GTTTGCTGATCATCGGGTCGTAGTATTC-3' | |
|  | **Primers for the qRT-PCR** | |
| *pykF*-F | 5'-GGTTTCGCAGCCGACCTAA-3' | 99% |
| *pykF*-R | 5'-AGTCAACGCCTTGCTCACA-3' |  |
| Vvrr1-F | 5'-TTTGAGGGCAATATCTCGAGTTT-3' | 103% |
| Vvrr1-R | 5'-TGTTGATAGCGGTGTTACCCAA-3' |  |
| *VMC_05940*-F | 5'-ACCATGATCGACGGTGGTTT-3' | 97% |
| *VMC_05940*-R | 5'-TGGTACTAGCTGCCACCAAA-3' |  |
| *frdC*-F | 5'-AAAGGCAACACCATCAAACACC-3' | 99% |
| *frdC*-R | 5'-CAGACGCACATTCACCCACC-3' |  |
| *VMC_15210*-F | 5'-GAAGGCGGTAACCTAATCGT-3' | 101% |
| *VMC_15210*-R | 5'-TGTCTTGACCAGCGTAAGTGT-3' |  |
| *VMC_28520*-F | 5'-TGGAATATTAAGAGGTCAACA-3' | 99% |
| *VMC_28520*-R | 5'-TGAGGTAATGGAGAAAACA-3' |  |
| *sdhB*-F | 5'-CCTTACATGAAAGAGTATACCCTTG-3' | 99% |
| *sdhB*-R | 5'-TATCATAGAACTGCGTCATGTCAAC-3' |  |
| *eno*-F | 5'-CCTCTATACGAGCACATCGC-3' | 103% |
| *eno*-R | 5'-AACCACCTTCATCACCAACT-3' |  |
| *ndk*-F | 5'-TTCTTGAGCCAGAACAAATG-3' | 101% |
| *ndk*-R | 5'-TGGTATGTGATATCGACAATTTC-3' |  |
| *glpF*-F | 5'-AGCCCATTGTCTGCTGTACCTTT-3' | 97% |
| *glpF*-R | 5'-CACTCTTGTAGGCACCGCTAACC-3' |  |
| *glpT*-F | 5'-GGGCAACCCAACAGTAGATATG-3' | 99% |
| *glpT*-R | 5'-GAAGTAATACAAGAACCCACTAAGA-3' |  |
| *VMC_28550*-F | 5'-GTGGGGAAAGTTACGCCTAAAAATA-3' | 98% |
| *VMC_28550*-R | 5'-ATCGGCACTGGACGTGCTTC-3' |  |
| *VMC_30580*-F | 5'-GCTTACAATCTGCACTGGATATTTC-3' | 97% |
| *VMC_30580*-R | 5'-TAGTTTCAATCCAATCTTCTAACGC-3' |  |
| *cysD*-F | 5'-AGAAATCACGGGCGAAAGAG-3' | 96% |
| *cysD*-R | 5'-ACAACCAAGGGTACGGAAGC-3' |  |
| *cysH*-F | 5'-AATCTCGTGCAGGTTTACCG-3' | 101% |
| *cysH*-R | 5'-TCAGAGCCATCATCTTCGTGT-3' |  |
| *gyrB*-F | 5'-TTGATTTACACTCCTCGCCTAAC-3' | 99% |
| *gyrB*-R | 5'-CAGCCAACTTGCCGTACTCT-3' |  |
