# Supplementary material for: Mechanisms underlying the virulence regulation of new Vibrio alginolyticus ncRNA Vvrr1 with a comparative proteomic analysis
Source: Emerg Microbes Infect. 2019 Nov 12;8(1):1604–18. doi: 10.1080/22221751.2019.1687261 (PMC6853220; doi:10.1080/22221751.2019.1687261)
Supplement: Supplemental Material [file TEMI_A_1687261_SM9120.zip › Supplementary Material/Table S2.docx]

| Table S2. Differentially expressed proteins | | | | | |
| --- | --- | --- | --- | --- | --- |
| Protein | **log_2_FC** | ***P*-value** | **Protein** | **log_2_FC** | ***P*-value** |
| CadA | 1.84 | 0.0282 | CheY | -0.58 | 0.0203 |
| CadB | 1.34 | 0.0167 | N646_2636 | -0.58 | 0.0040 |
| PyrH | 1.30 | 0.0020 | SdhA | -0.58 | 0.0006 |
| Ocd | 1.27 | 0.0239 | N646_1925 | -0.58 | 0.0236 |
| N646_4243 | 1.27 | 0.0155 | NrfB | -0.58 | 0.0032 |
| N646_3688 | 1.25 | 0.0086 | Eno | -0.59 | 0.0019 |
| MalP | 1.25 | 0.0021 | GDE1 | -0.59 | 0.0031 |
| CbbBc | 1.17 | 0.0010 | HutX | -0.59 | 0.0017 |
| FadL | 1.16 | 0.0075 | RacD | -0.59 | 0.0146 |
| N646_4596 | 1.15 | 0.0040 | N646_4501 | -0.59 | 0.0007 |
| FocA_3 | 1.09 | 0.0030 | NtrB | -0.59 | 0.0044 |
| FrdC | 1.09 | 0.0037 | CstA | -0.59 | 0.0335 |
| K08M4_12250 | 1.08 | 0.0082 | N646_3241 | -0.59 | 0.0007 |
| K08M4_08940 | 1.06 | 0.0335 | AL539_20280 | -0.59 | 0.0031 |
| AOG25_03345 | 1.06 | 0.0096 | QcrA | -0.60 | 0.0013 |
| LamB | 1.04 | 0.0245 | AL539_16120 | -0.60 | 0.0000 |
| AdhE | 1.04 | 0.0013 | SstT | -0.60 | 0.0010 |
| AhpC | 1.02 | 0.0012 | N646_2511 | -0.60 | 0.0080 |
| FrdB | 1.01 | 0.0219 | N646_4560 | -0.61 | 0.0171 |
| MalK | 0.98 | 0.0106 | AOG25_19225 | -0.61 | 0.0024 |
| BioD | 0.93 | 0.0021 | GltP | -0.61 | 0.0121 |
| FdoI | 0.91 | 0.0108 | Ppa | -0.61 | 0.0004 |
| AT730_06230 | 0.91 | 0.0068 | K04M1_47200 | -0.61 | 0.0004 |
| K04M1_09410 | 0.88 | 0.0075 | SDH | -0.61 | 0.0004 |
| Can | 0.86 | 0.0069 | RplQ | -0.62 | 0.0452 |
| GloA_2 | 0.86 | 0.0000 | AOG25_03130 | -0.62 | 0.0369 |
| N646_3560 | 0.83 | 0.0177 | N646_2482 | -0.63 | 0.0003 |
| FtsK | 0.82 | 0.0423 | N646_1001 | -0.63 | 0.0016 |
| MenC | 0.80 | 0.0435 | VMC_30580 | -0.63 | 0.0411 |
| N646_4365 | 0.79 | 0.0011 | Pgm | -0.64 | 0.0001 |
| ACS86_07865 | 0.79 | 0.0035 | GST | -0.64 | 0.0001 |
| Pygl-1 | 0.78 | 0.0043 | AL539_14515 | -0.64 | 0.0025 |
| PtsG | 0.77 | 0.0042 | RpmH | -0.64 | 0.0175 |
| AhpF | 0.77 | 0.0004 | N646_0592 | -0.65 | 0.0090 |
| FrdD | 0.75 | 0.0076 | Asd1 | -0.65 | 0.0067 |
| VMC_15210 | 0.74 | 0.0095 | CBS | -0.65 | 0.0000 |
| FrdA | 0.73 | 0.0137 | AlgE7 | -0.65 | 0.0053 |
| SSR | 0.71 | 0.0355 | N646_3142 | -0.65 | 0.0062 |
| N646_4363 | 0.71 | 0.0075 | CysK | -0.65 | 0.0001 |
| SecF | 0.69 | 0.0030 | HutU | -0.65 | 0.0005 |
| SecD | 0.68 | 0.0008 | PurM | -0.66 | 0.0001 |
| DcuC | 0.68 | 0.0042 | AOG25_05755 | -0.66 | 0.0014 |
| DmsA | 0.67 | 0.0318 | N646_1091 | -0.66 | 0.0001 |
| PflB | 0.66 | 0.0056 | OmpA | -0.67 | 0.0102 |
| RpoZ | 0.66 | 0.0448 | AL539_22720 | -0.67 | 0.0013 |
| DcuA | 0.66 | 0.0322 | N646_2542 | -0.67 | 0.0007 |
| MalK | 0.66 | 0.0295 | N646_3390 | -0.67 | 0.0010 |
| ArtI | 0.65 | 0.0293 | HpaF | -0.68 | 0.0021 |
| AL539_00785 | 0.65 | 0.0023 | K04M1_41120 | -0.68 | 0.0001 |
| Tal | 0.64 | 0.0009 | Ndk | -0.68 | 0.0000 |
| AL539_20350 | 0.64 | 0.0094 | YdiY | -0.69 | 0.0002 |
| AL539_25870 | 0.63 | 0.0104 | VMC_28550 | -0.69 | 0.0026 |
| N646_1365 | 0.63 | 0.0375 | PykF | -0.70 | 0.0000 |
| MalF | 0.63 | 0.0293 | N646_4204 | -0.70 | 0.0006 |
| AOG25_23770 | 0.63 | 0.0428 | N646_0601 | -0.70 | 0.0043 |
| Cdd | 0.62 | 0.0009 | GuaC | -0.70 | 0.0001 |
| AL539_11945 | 0.62 | 0.0113 | MsrA | -0.70 | 0.0001 |
| Crr | 0.62 | 0.0077 | AOG25_06795 | -0.71 | 0.0166 |
| AOG25_25510 | 0.61 | 0.0004 | N646_1261 | -0.71 | 0.0011 |
| N646_3913 | 0.61 | 0.0162 | ACS86_00920 | -0.71 | 0.0463 |
| Pgk | 0.59 | 0.0015 | N646_2994 | -0.71 | 0.0001 |
| AOG25_15680 | 0.59 | 0.0124 | GlpD | -0.71 | 0.0078 |
| N646_4210 | 0.59 | 0.0080 | AOG25_18310 | -0.72 | 0.0000 |
| Rne | 0.59 | 0.0076 | PetB | -0.72 | 0.0012 |
| SthA | -0.72 | 0.0002 | SucA | -0.93 | 0.0000 |
| VMC_23910 | -0.72 | 0.0108 | VMC_10930 | -0.94 | 0.0001 |
| GcvH | -0.73 | 0.0028 | HutH | -0.94 | 0.0003 |
| SdhA | -0.74 | 0.0010 | N646_3000 | -0.95 | 0.0000 |
| N646_0290 | -0.74 | 0.0009 | Mdh1 | -0.96 | 0.0002 |
| N646_1092 | -0.74 | 0.0000 | GlpK | -0.97 | 0.0010 |
| AL539_16760 | -0.75 | 0.0000 | CysC | -0.98 | 0.0005 |
| CysK_2 | -0.75 | 0.0000 | IDH1 | -0.98 | 0.0000 |
| K04M1_00360 | -0.76 | 0.0002 | YclQ | -1.00 | 0.0005 |
| K05K4_16210 | -0.77 | 0.0016 | GlyA | -1.00 | 0.0000 |
| AL539_09960 | -0.77 | 0.0001 | N646_4714 | -1.00 | 0.0000 |
| SucC | -0.77 | 0.0212 | AstD | -1.01 | 0.0000 |
| RibB | -0.80 | 0.0002 | Tdh | -1.02 | 0.0000 |
| AbgT_2 | -0.80 | 0.0023 | CysJ | -1.06 | 0.0000 |
| AL539_00075 | -0.81 | 0.0003 | CysH | -1.06 | 0.0000 |
| VMC_31200 | -0.81 | 0.0000 | AOG25_14795 | -1.06 | 0.0001 |
| ACS86_17285 | -0.81 | 0.0009 | ACS86_16920 | -1.06 | 0.0000 |
| Fbp | -0.82 | 0.0001 | GlpF | -1.08 | 0.0003 |
| CysI | -0.82 | 0.0000 | ArcA | -1.09 | 0.0000 |
| N646_1886 | -0.82 | 0.0000 | CysN | -1.09 | 0.0001 |
| N646_1090 | -0.83 | 0.0001 | AT730_12740 | -1.10 | 0.0001 |
| AstA | -0.83 | 0.0009 | E1.2.1.88 | -1.12 | 0.0000 |
| AL539_10400 | -0.85 | 0.0000 | N646_3437 | -1.14 | 0.0000 |
| N646_1948 | -0.85 | 0.0017 | Kbl | -1.15 | 0.0001 |
| N646_2655 | -0.85 | 0.0067 | N646_4712 | -1.15 | 0.0000 |
| CTBS | -0.85 | 0.0154 | CysD | -1.16 | 0.0001 |
| VMC_36650 | -0.86 | 0.0001 | Hgd | -1.19 | 0.0000 |
| DppB_1 | -0.86 | 0.0210 | N646_0293 | -1.19 | 0.0026 |
| CysK | -0.86 | 0.0000 | SucD | -1.21 | 0.0003 |
| HutZ | -0.87 | 0.0009 | AruC | -1.21 | 0.0000 |
| Cc4_1 | -0.87 | 0.0002 | AL539_03155 | -1.22 | 0.0000 |
| N646_2188 | -0.89 | 0.0001 | SucC | -1.23 | 0.0000 |
| GDE1 | -0.89 | 0.0164 | AsnB | -1.23 | 0.0002 |
| N646_0605 | -0.89 | 0.0016 | GlpK | -1.27 | 0.0002 |
| ZapE | -0.89 | 0.0009 | K04M1_02370 | -1.28 | 0.0002 |
| FutA1 | -0.90 | 0.0008 | RstB _2 | -1.31 | 0.0000 |
| GlpQ | -0.90 | 0.0035 | AsnB | -1.39 | 0.0000 |
| SdhB | -0.90 | 0.0012 | AOG25_17495 | -1.42 | 0.0049 |
| AcnB | -0.91 | 0.0002 | AtpE | -1.44 | 0.0005 |
| GcvP | -0.91 | 0.0000 | FucA | -1.51 | 0.0000 |
| IdnT | -0.91 | 0.0006 | AT730_15715 | -1.55 | 0.0000 |
| GlpT | -0.91 | 0.0003 | N646_0608 | -1.56 | 0.0001 |
| N646_0629 | -0.91 | 0.0002 | RstB1 | -1.59 | 0.0005 |
| FbpA | -0.91 | 0.0018 | Gap | -1.62 | 0.0023 |
| AL539_00825 | -0.91 | 0.0003 | Ald | -1.65 | 0.0000 |
|  |  |  | N646_4711 | -2.22 | 0.0000 |
