# Supplementary figures and images for: Mechanisms underlying the virulence regulation of new Vibrio alginolyticus ncRNA Vvrr1 with a comparative proteomic analysis
Source: Emerg Microbes Infect. 2019 Nov 12;8(1):1604–18. doi: 10.1080/22221751.2019.1687261 (PMC6853220; doi:10.1080/22221751.2019.1687261)

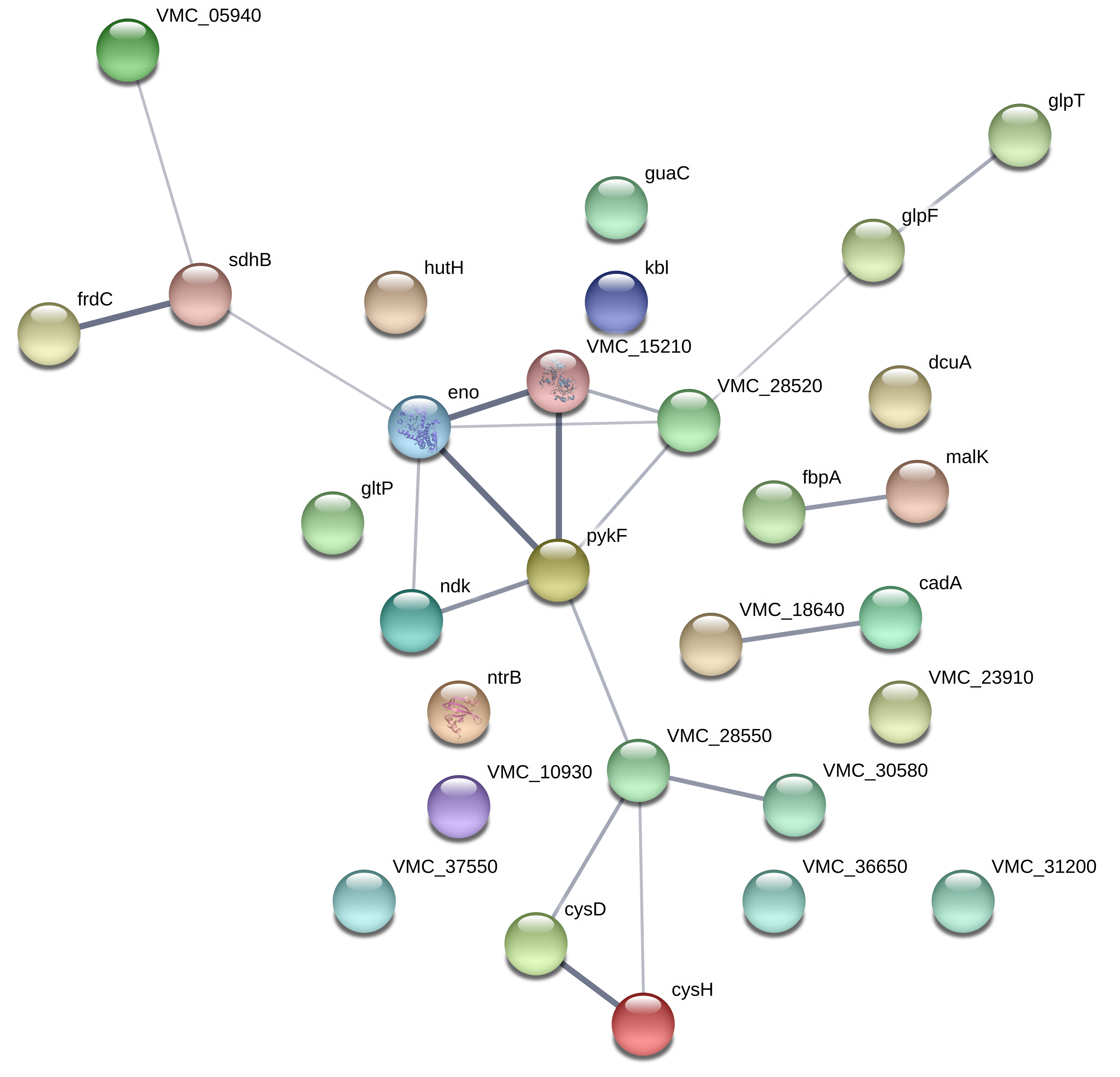

Supplement: Supplemental Material [file TEMI_A_1687261_SM9120.zip › Supplementary Material/Figure S1.tif]

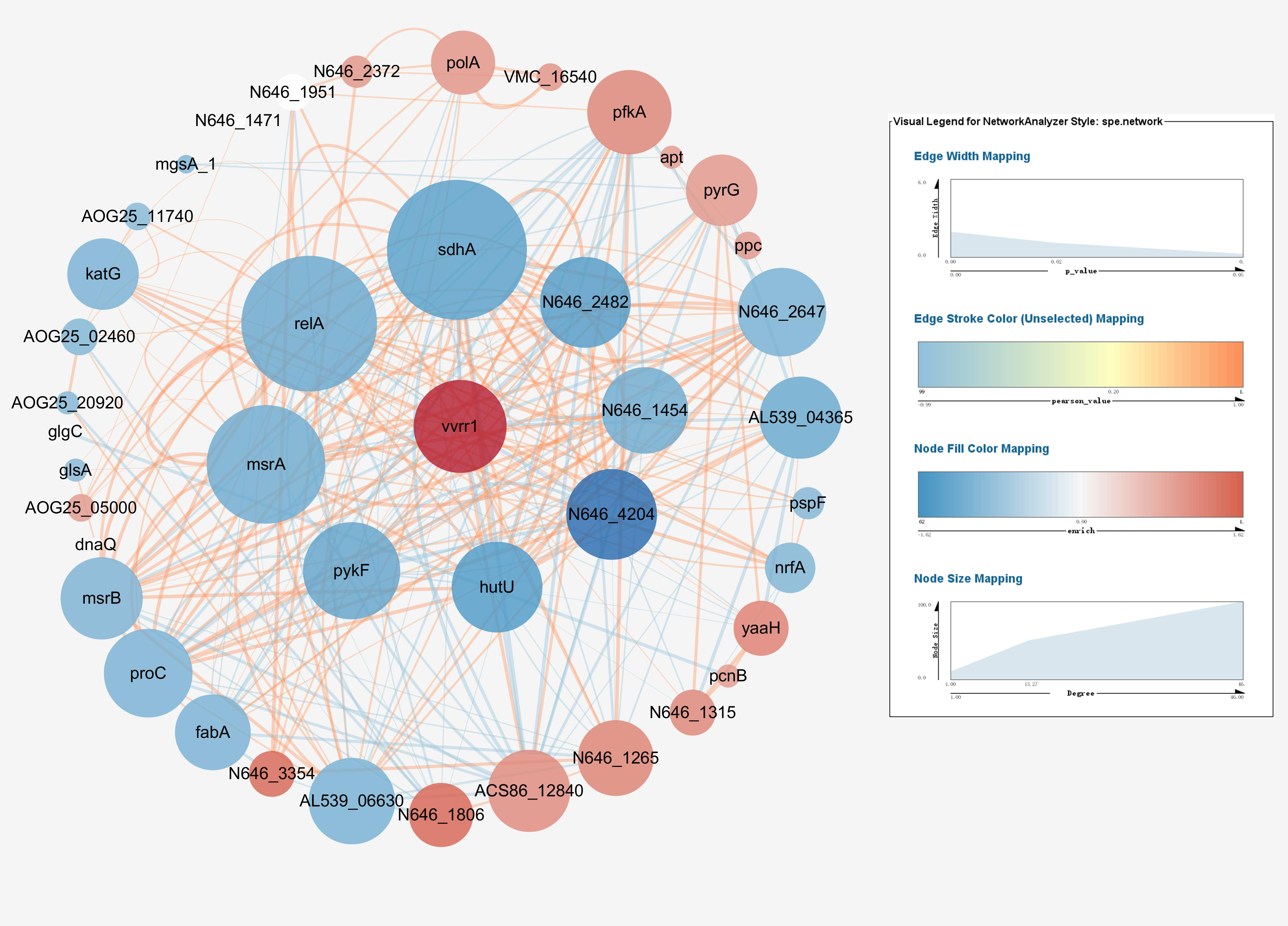

Supplement: Supplemental Material [file TEMI_A_1687261_SM9120.zip › Supplementary Material/Figure S2.tif]
